# UniPolar Radiofrequency for Treatment of Facial Aging Signs and Skin Laxity

**DOI:** 10.1111/jocd.71102

**Published:** 2026-07-27

**Authors:** Krenar Dobroshi, Iva Stoilova, Bekim Ismaili, Marija Glavash Dodov, Renata Slaveska Raichki

**Affiliations:** ^1^ Alma Mater Europaea College of Medical Sciences “Rezonanca” Prishtina Kosovo; ^2^ Aesthavist Laser & Dermatology Clinic Sofia Bulgaria; ^3^ University of Tetovo Tetovo North Macedonia; ^4^ The Saints Cyril and Methodius University Skopje North Macedonia

**Keywords:** aging, radiofrequency, skin laxity, skin tightening, unipolar, wrinkles

## Abstract

**Background:**

Radiofrequency (RF) technologies are widely used for noninvasive skin rejuvenation. Unlike other RF technologies, unipolar RF emits an electromagnetic field that produces controlled heat within the adjacent area, promoting collagen remodeling and improving the visible signs of skin aging.

**Objective:**

This study evaluated the safety and efficacy of unipolar RF in improving facial skin laxity and wrinkles.

**Methods:**

A retrospective analysis was conducted on *n* = 93 patients treated with a unipolar RF applicator. Clinical outcomes were assessed using the Global Aesthetic Improvement Scale (GAIS) and Modified Fitzpatrick Wrinkle and Elastosis Scale (MFWS). Blinded evaluators compared pre‐ and posttreatment photographs, and patient‐reported outcomes, treatment‐associated pain, and overall satisfaction were also analyzed.

**Results:**

GAIS showed overall improvement (mean score 1.9 ± 0.5) and MFWS values decreased following treatments (mean change −0.3 ± 0.3; *p* < 0.001). A significant negative correlation between GAIS and MFWS reduction (*p* < 0.001) as well as a positive correlation for higher treatment numbers and GAIS outcome were found. Patients rated their satisfaction as “satisfied” to “much satisfied.” No serious adverse events were reported.

**Conclusion:**

Unipolar radiofrequency demonstrated significant improvements in facial skin laxity and wrinkles with minimal discomfort and a high safety profile.

**Abbreviations**
EMR: Electromagnetic radiation
GAIS: Global Aesthetic Improvement Scale
MFWS: Modified Fitzpatrick Wrinkle and Elastosis Scale
RF: Radio frequency

## 1 Introduction

Skin aging is characterized by collagen and elastin degradation, as well as dermal and epidermal thinning and fibroblast dysfunction [1, 2, 3]. Common signs of aging include changes in pigmentation, sagging, telangiectasia, and wrinkling [2]. These processes are driven by intrinsic factors, including chronological aging and genetics, and by extrinsic environmental exposures, such as sun and smoking, as well as mental stress, diet, work habits, drug abuse, and disease [2, 3, 4].

In recent years, a steady increase in aesthetic procedures has been observed worldwide [5]. Specifically, demand for skin rejuvenation has grown rapidly and is projected to reach $44.5 billion annually by 2030 [3]. Skin rejuvenation, aiming to restore the balanced facial fullness that characterizes a youthful appearance, can be achieved in several ways, with current trends shifting from excision and suspension procedures to less invasive approaches [4]. Among noninvasive aesthetic procedures, radiofrequency (RF) is considered effective for reducing wrinkles and tightening skin laxity [6, 7].

RF delivers electric energy transferred into heat (dielectric heating) into the dermis and subdermal layers to remodel collagen and elastin [8] RF modules can be monopolar, bipolar, unipolar, multipolar and fractional [9, 10] They differ mainly in the way their energy is delivered [11]. Monopolar, bipolar and multipolar are using an electric current penetrating body structure to an opposing electrode. Monopolar RF facilitates deeper penetration than unipolar and bipolar RF, with current flowing from a single active electrode through the skin to a grounding pad [11]. Bipolar RF generates the electric current between two neighboring electrodes which allow controlled, more superficial heating of the skin [12].

The Unipolar RF applicator used in this study emits omnidirectional electromagnetic radiation (EMR) without the need for a grounding pad. The penetration depth of unipolar RF reaches about 15–20 mm on the level of subcutaneous tissue and was found safe and effective for treating skin laxity, wrinkles, and cellulite in previous studies [10, 13, 14, 15]. In this study, the unipolar RF applicator uses a unique high radiofrequency of 40.68 MHz allowing deep, homogenous heating for more uniform treatment results.

The purpose of this study was to evaluate the safety and efficacy of a noninvasive, unipolar RF treatment for skin tightening and rejuvenation in patients presenting skin laxity and facial wrinkles. Using a retrospective real‐world clinical study design, the investigation aimed to validate previously reported outcomes and further demonstrate the effectiveness of treatment in improving signs of facial aging.

## 2 Methods

**FIGURE 1:**
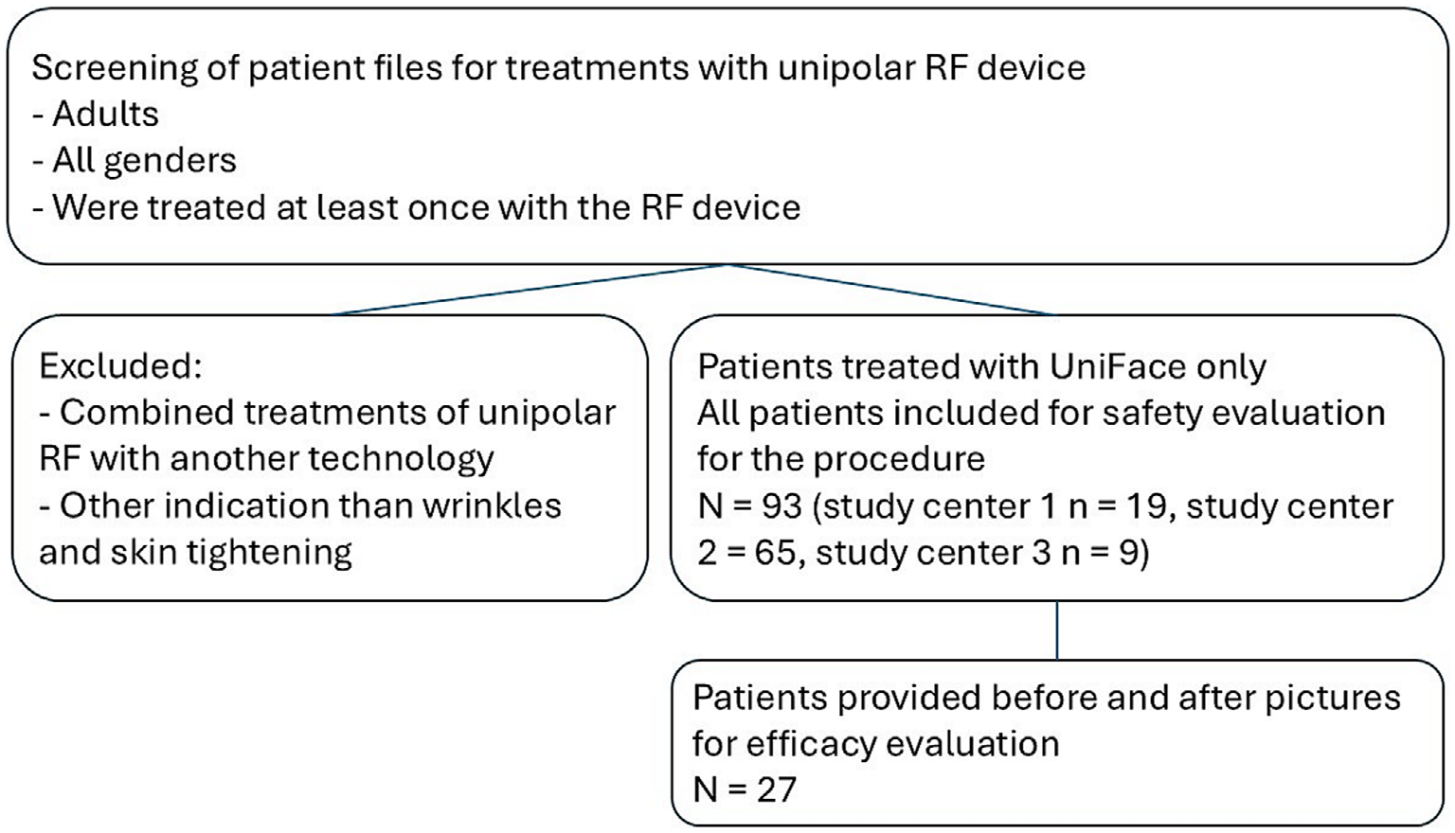
Flowchart to select suitable patients to be included in the analysis.

In this retrospective cohort study, clinical data from *n* = 93 patients treated for facial skin laxity and wrinkles with Accent Prime system between 2023 and 2025 were analyzed. The study protocol was approved by the local ethics committee. In accordance with routine clinical practice, all patients provided written informed consent prior to treatment, permitting the anonymous use of their clinical data for scientific evaluation. Patient records were systematically screened, and eligible cases meeting the study criteria were included in the analysis (Figure 1).

### 2.1 Study Population

Patients were included if they had undergone at least one treatment with the RF device for facial laxity or wrinkles. All genders and treatment conditions were included. Exclusion criteria were the general contraindications for RF treatments such as current or recent use of oral isotretinoin, presence of an active autoimmune skin disorder, pregnancy, breastfeeding. Additional exclusion criteria specific to this study were absence of informed consent to evaluate the patient photographs and receipt of other skin laxity treatments, such as fractional or ablative laser, injectables, surgery, or chemical peeling within 3 months before treatment, during treatment course, or within 3 months after treatment.

Patient‐ and treatment‐specific variables collected included gender, age, smoking status, Fitzpatrick skin type, number of treatments, patient‐specific treatment protocol and treatment intervals (weeks). Safety was evaluated by documented adverse events in patient's records during treatment and follow‐up visits, and patient‐reported pain scores, assessed on a 10‐point scale (1 = low, 10 = high).

### 2.2 Study Procedure

All treatments were performed using the Accent Prime (Alma Lasers, Caesarea, Israel) with the UniFace non‐ablative unipolar RF applicator intended to provide volumetric deep tissue heating for aesthetic improvement. Prior to each treatment, the target area was thoroughly cleansed and dried. During the procedure, skin temperature was continuously monitored to maintain a target range of 40°–42°. Simultaneously, the RF tip was actively cooled using integrated thermoelectric cooling (TEC) technology to enhance patient comfort and protect the epidermis. For facial treatments, power settings ranged from 65 to 115 W, with total accumulated energy levels between 30 and 70 kJ per half‐face. For the forehead, power settings ranged from 60 to 100 W, with accumulated energy levels between 30 and 50 kJ. Treatments were performed using an in‐motion technique to ensure uniform energy delivery across the treatment area.

### 2.3 Outcome Measures

Treatment efficacy was assessed using multiple outcome measures:

1. Mean Blinded Global Aesthetic Improvement Scale (GAIS): Three independent, blinded evaluators assessed treatment outcomes using the GAIS 5‐point scale (0 = worse, 1 = no change, 2 = mild improvement, 3 = much improvement, 4 = very much improvement). Mean blinded GAIS scores were calculated as the average of the three independent evaluator assessments. The final posttreatment photographs were obtained 2–3 weeks after completion of the last treatment session
2. Mean Modified Fitzpatrick Wrinkle and Elastosis Scale (MFWS) Change: Wrinkle severity was assessed by the same three blinded evaluators using the MFWS with seven graded classes ranging from Class 0 (no wrinkles) to Class 3 (deep wrinkles) based on estimated wrinkle depth [16]. Mean change was calculated as the difference between posttreatment and pretreatment scores, where negative values indicated improvement in wrinkle severity
3. Physician GAIS: Non‐blinded assessment performed by the treating physician using the same 5‐point GAIS scale as detailed in outcome #1
4. Mean VISIA score change: The VISIA Complexion Analysis Camera (Canfield Scientific, USA) captured standardized images to quantify wrinkle features using absolute scores, which measure the size, area, and intensity of skin features. Changes from baseline were calculated as the average of bilateral measurements, where negative change scores indicate clinical improvement [17, 18]
5. Patient Satisfaction: Self‐reported on a 5‐point Likert scale (0 = worse, 1 = no change, 2 = satisfied, 3 = much satisfied, 4 = very much satisfied)

Due to the retrospective design of the study, outcome measure availability varied across patient population, with some having incomplete assessment records. The unified analytical approach optimized use of available data while accounting for missing observations and differences in measurement scales.

### 2.4 Statistical Methods

Patient characteristics and outcome measures were summarized using descriptive statistics. Continuous variables are presented as means and standard deviations (SD), and categorical variables as frequencies and percentages. To accommodate multiple outcome types with varying measurement scales, the dataset was restructured from wide to long format. This transformation enabled unified analysis of all outcome measures as continuous variables, including mean evaluator scores and within‐subject change scores, while accounting for scale differences through covariate adjustment. Outcome values were modeled as a function of outcome type and potential predictors, including age (centered on the sample mean), number of treatments, smoking status, and treatment intervals. Outcome type was included as a covariate to account for scale differences between measurement methods. Linear mixed‐effects models with patient‐level random intercepts were initially fitted to account for within‐patient correlation across multiple outcomes. When random intercept variance was not significant, indicating minimal between‐patient variability, pooled linear regression was used instead. Patient‐level bootstrap (1000 replications) was employed to account for within‐patient clustering and provide robust inference, with 95% confidence intervals derived from the bootstrap distributions. Correlations between outcome measures were computed to assess consistency between mean blinded GAIS and each other outcome measure. These associations were based on available pairwise data; therefore, sample sizes varied across correlations and were smaller than the full analysis sample. Nonparametric tests were used where appropriate. Model assumptions were verified through diagnostic assessment. Statistical significance was set at α = 0.05. All analyses were performed using R statistical software (version 4.3.3).

### 2.5 Safety Assessment

Adverse events and patient‐reported pain were summarized using frequencies and percentages.

## 3 Results

The study population included *n* = 93 patients with a mean age of 44.5 ± 11.4 years. The majority were female (93.5%) and nonsmokers (71.0%). Most patients had Fitzpatrick skin types II (54.8%) and III (41.9%). On average, patients underwent 2.0 ± 1.2 treatment sessions, ranging from 1 to 6 sessions in total. Specifically, *n* = 45 patients received one treatment, *n* = 17 received two treatments, *n* = 15 received three treatments, *n* = 12 received four treatments, and *n* = 2 patients each underwent five and six treatments, respectively. The mean interval between sessions was 2 ± 0.7 weeks. Standardized pre‐ and posttreatment photographs suitable for blinded evaluation were available for *n* = 27 patients. This subgroup underwent an average of 3.0 ± 1.01 treatment sessions (range: 2–5), with a mean treatment interval of 2 ± 1.6 weeks. Detailed demographic and treatment characteristics are summarized in Table 1.

**TABLE 1:** Patients and treatment characteristics.

Characteristic	*n* = 93
Age, years, mean ± SD (range)	44.5 ± 11.4 (17–79)
Gender, *n* (%)	
Female	87 (93.5%)
Male	6 (6.5%)
Smoking, *n* (%)	
No	66 (71%)
Yes	24 (25.8%)
Fitzpatrick skin type, *n* (%)	
Type I	1 (1.1%)
Type II	51 (54.8%)
Type III	39 (41.9%)
Type IV	2 (2.2%)
Number of treatments, mean ± SD (range)	2 ± 1.2 (range 1–5 treatments)
Treatment intervals weeks, mean ± SD (range)	2 ± 0.7 (range 1–6 weeks)

**FIGURE 2:**
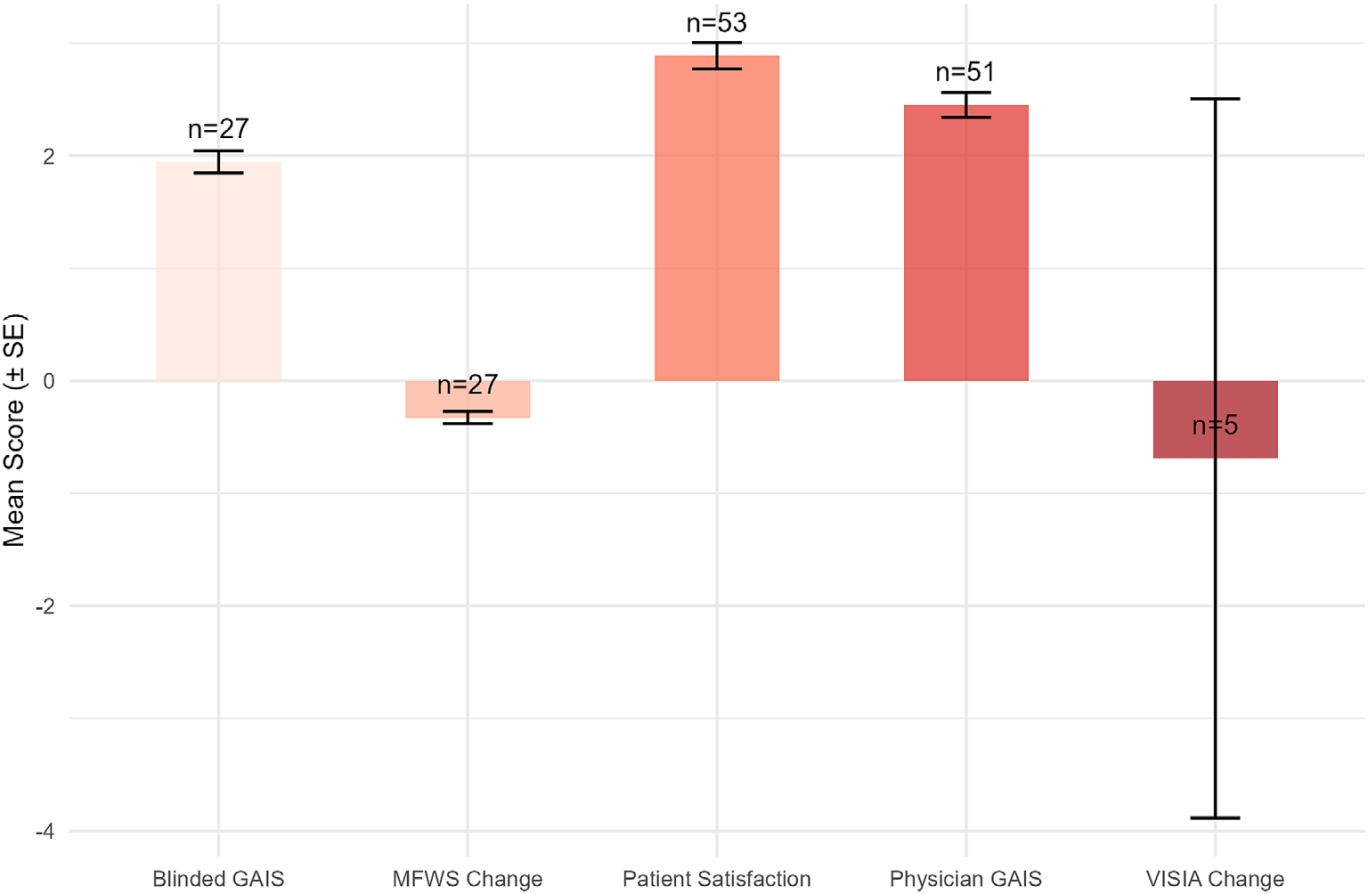
Mean improvement by outcome measure—blinded GAIS, MFWS, Patient satisfaction, physician GAIS and VISIA change. Scales differ across measures, with patient groups overlapping but not identical.

The mean blinded assessment using GAIS was 1.9 ± 0.5 (*n* = 27), indicating overall visible aesthetic improvement following treatment (Figure 2). The blinded MFWS change was −0.3 ± 0.3 (*n* = 27), indicating a reduction in wrinkle severity. This improvement was statistically significant (Wilcoxon signed‐rank test, *p* < 0.001). The non‐blinded physician‐rated GAIS was 2.5 ± 0.8 (*n* = 51), supporting the favorable clinical outcomes observed in the blinded evaluation. VISIA wrinkle analysis, available for only five patients, showed a mean score reduction of −0.7 ± 7.1, suggesting a trend toward improvement despite the high variability in between the five patients. Patient satisfaction was high, with a mean score of 2.9 ± 0.8 (*n* = 53). A summary of clinical outcome measures is presented in Table 2.

**TABLE 2:** Descriptive statistics of outcome measures.

Outcome	*N*	Mean ± SD	Range
Mean blinded GAIS	27	1.9 ± 0.5	1 to 2.8
Mean blinded MFWS difference	27	−0.3 ± 0.3	−0.8 to 0.3
Physician GAIS	51	2.5 ± 0.8	1 to 4
Mean VISIA (wrinkle score) difference	5	−0.7 ± 7.1	−13.3 to 4.4
Patient satisfaction	53	2.9 ± 0.8	1 to 4

### 3.1 Treatment Efficacy

As presented in Figure 2, blinded GAIS assessments on patients with available before and after treatment photographs (*n* = 27) demonstrate that 78% of patients experienced visible improvement, with 18.5% achieving “much improvement”.

**FIGURE 3:**
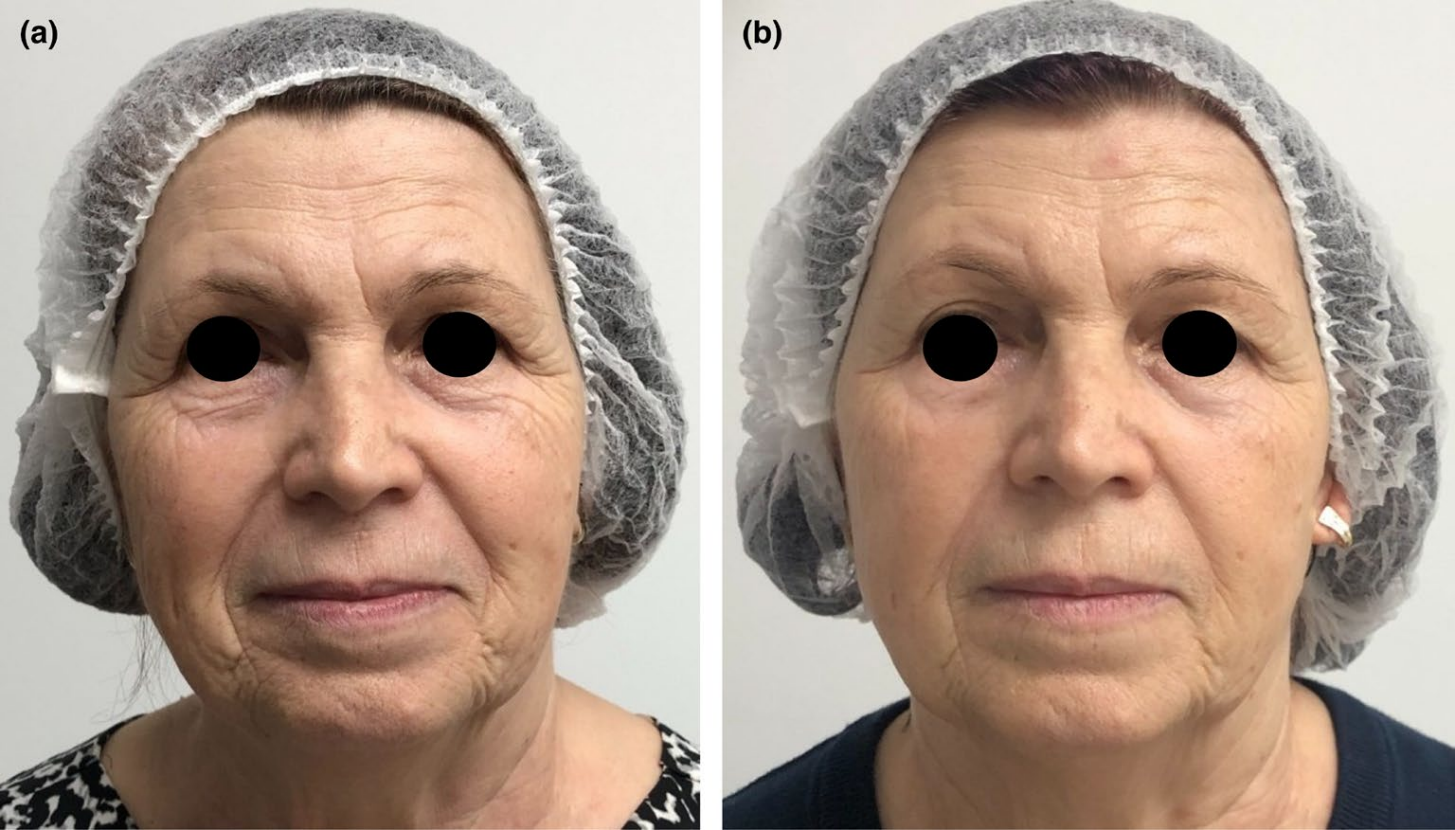
Representative patient before (a) and after (b) treatment. Female patient showed improved skin laxity and lower wrinkle severity compared to before and after treatment.

**FIGURE 4:**
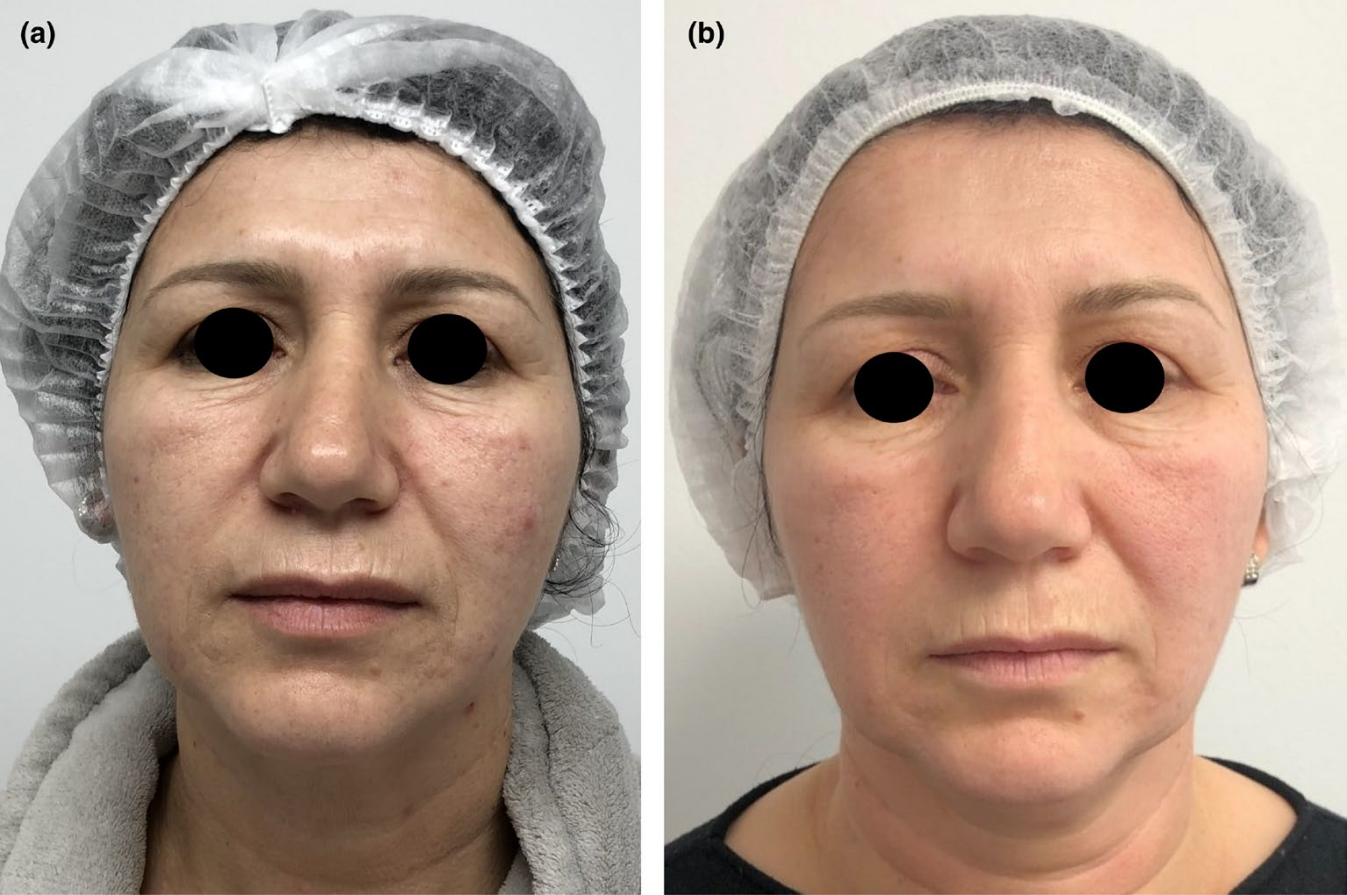
Representative patient before (a) and after (b) treatment. Female patient showed improved skin laxity and lower wrinkle severity compared to before and after treatment.

Representative images of treated patients are displayed in Figures 3 and 4. Figure 2 summarizes the mean scores across all evaluated outcome measures. For blinded GAIS, physician GAIS, and patient satisfaction, higher scores indicate better outcomes, while for blinded MFWS change and VISIA change, lower scores reflect improvement. All outcome measures consistently demonstrated favorable improvement following treatment. Standard error (SE) bars for VISIA wrinkle analysis were comparatively wider, reflecting the lower statistical precision associated with the smaller sample size (*n* = 5).

### 3.2 Treatment Predictors

The initial mixed‐effects model included outcome type, age, number of treatments, smoking status, and treatment intervals as fixed effects, with patient‐specific random intercepts. Gender and skin type were excluded due to insufficient variability (93.5% female; 96.7% skin types II‐III). In this model, the patient‐level random intercept was not statistically significant, suggesting minimal residual variation between patients after accounting for fixed effects. We therefore proceeded with pooled linear regression using the same predictor structure and bootstrap method to provide robust inference.

Using a unified model analyzing all outcome measures while controlling scale differences, the pooled analysis revealed a significant positive association between the number of treatments and clinical outcome (β = 0.23, 95% CI: 0.048–0.576, *p* = 0.014). Each additional treatment increases outcome scores by 0.23 points on average. Patient age, smoking status, and treatment intervals did not reach statistical significance.

### 3.3 Correlations Analyses

Pairwise correlation analysis using Spearman's rank method showed a strong negative correlation between blinded MFWS change and blinded GAIS (*r* = −0.716, *p* < 0.001), indicating good agreement between the two blinded assessments, as MFWS uses reverse scoring (negative values reflect improvement). A positive correlation between blinded GAIS and treatment numbers was found (*r* = 0.392, *p* = 0.059), which was not statistically significant. In contrast, both physician‐rated GAIS (*r* = −0.499, *p* = 0.013) and patient satisfaction (*r* = −0.386, *p* < 0.051) were inversely correlated with blinded GAIS, suggesting perceptual differences between objective evaluator ratings and subjective assessments.

### 3.4 Safety

Treatment associated discomfort was assessed using a 0–10 numerical scale. Pain assessment data was available for *n* = 51 patients (54.8%), with a mean pain score of 1.4 ± 1.5, indicating that the procedure was generally well tolerated and associated with minimal discomfort during treatment.

The most commonly reported treatment related reaction was transient erythema, observed in *n* = 65 patients (69.9%) that resolved without intervention. Mild redness was reported in 1 patient (1.1%). No treatment‐related reactions were reported in *n* = 27 patients (29%). No adverse events were reported throughout the study period.

## 4 Discussion

This study demonstrated that the unipolar RF handpiece for facial skin tightening and wrinkle treatment was well tolerated and associated with positive clinical outcomes. According to three blinded evaluators using the GAIS scale, the majority of patients experienced improvement, with one in five patients showing substantial improvement. Based on the MFWS, the blinded evaluators observed a statistically significant reduction in wrinkle severity following treatment. Correlation analysis indicated concordance between blinded MFWS and GAIS assessments. While some divergence was noted between blinded evaluator scores, physician ratings, and patient‐reported outcomes (likely reflecting differences in expectation versus objective changes), overall satisfaction remained high. Nonetheless, the mean patient‐reported satisfaction scores demonstrated that patients were highly satisfied with their treatment outcomes. The treatment was effective across diverse patient demographics, with only minimal discomfort reported during procedures, and temporary erythema being the most common adverse effect.

Aged skin is characterized by increased roughness and loss of elasticity. Type I collagen, which forms triple‐helix structures from three polypeptide chains, undergoes contradictory changes. While its density increases, weakened cross‐links compromise its structural integrity. The deterioration of dermal elastic fibers and the loss of subcutaneous fat contribute to the appearance of laxity with age [19]. Therefore, to improve wrinkles and lax skin, it is crucial to target the deep skin layer and subcutaneous tissue [11, 20]. Over the past decade, RF has become increasingly popular for facial rejuvenation because of its minimal side effects and brief recovery time for patients [11]. All RF methods operate by heating tissues to trigger collagen denaturation, remodeling, contraction, and regeneration [11]. As dermal temperatures rise, the helical structure of collagen fibers is disrupted, causing them to contract immediately and creating a visible tightening effect [10, 11, 20, 21]. A wound‐healing cascade follows, during which fibroblasts are stimulated to initiate neocollagenesis and extracellular matrix remodeling [10, 20]. Importantly, throughout this process, the epidermis remains largely unaffected, making the procedure suitable for a variety of skin types [22]. Histological studies have shown temporary microinflammatory responses in fibroblasts following RF stimulation [11]. RF operates through electromagnetic waves, which cause ions and charged molecules to collide as they move through tissue, generating heat in deep cutaneous structures as the tissue resists this movement [20, 22, 23]. The depth of penetration is determined by tissue bioimpedance, lowest in water, then nerves, muscle, collagen, and fat, the type of RF, and the initial energy settings. At constant tissue impedance, RF penetration depth decreases as frequency increases, meaning lower frequencies reach deeper tissue layers more effectively [11]. Earlier devices had limited energy control, causing them to release energy superficially and indirectly heat the dermis. This affected the epidermis and prevented optimal collagen contraction [11, 20]. More recent modalities have improved both efficacy and safety [20]. Although the unipolar handpiece has been less studied than monopolar and bipolar devices [20], accumulating evidence demonstrates its efficacy and safety in reducing rhytids and improving skin laxity. Alexiades‐Armenakas et al. [24] conducted a randomized, blinded, split‐face study in *n* = 10 patients comparing mobile unipolar versus bipolar RF handpieces for rhytid reduction and skin laxity. Using a 4‐point comprehensive grading scale on split‐photographs, they showed mean improvements of 6.0% ± 4.6% in rhytid scores and 4.6% ± 4.8% in laxity scores on the unipolar‐treated side; however, which was not statistically significant. Treatments were described as painless and only transient erythema was reported. Zhang et al. [25] evaluated *n* = 52 patients with mild‐to‐moderate middle and lower facial laxity who underwent one to three monthly unipolar RF treatments. A blinded GAIS assessment concluded 75% were improved and 5.8% much improved. Suh et al. [20] evaluated *n* = 14 patients with mild‐to‐moderate facial laxity treated with a fractional unipolar RF device in five sessions at 2‐week intervals and followed the patients for 3 months posttreatment. The study reported 35.7% of subjects showed significant improvement, 50% moderate improvement, and 14.3% slight improvement of facial laxity using a 4‐point scale, with 85.7% patient satisfaction, and histologic analysis demonstrated an increase in thick collagen bundles in the reticular dermis without any serious adverse events. In a four‐patient case study, Cirillo‐Hyland [26] treated the face and neck with a unipolar RF handpiece, and all patients reported significant clinical improvement, and independent evaluators rated improvement between 40% and 65%. Previous studies vary in the number of treatment sessions, the intervals between treatments, the outcome assessment scales used, and the specific treatment parameters compared with the present study. Nevertheless, all reported studies demonstrated improvements in skin laxity and wrinkles following unipolar RF treatment. In the current study, a 78% improvement rate was observed, comparable to the 75% reported by Zhang et al., but lower than the 85.7% rate of moderate to significant improvement described by Suh et al. [20, 25]. This difference may be attributed to the shorter follow‐up period in the present study, as Suh et al. followed patients for 3 months, allowing neocollagenesis to become more apparent at a more advanced stage.

In this study using pooled analysis, treatment with an advanced unipolar RF handpiece provided evidence for positive treatment outcomes and a well‐tolerated treatment by the patients, producing reliable improvements in wrinkles and skin laxity. High concordance between two blinded assessment methods further supports these outcomes. This finding may reflect cohort heterogeneity or evaluation bias in the unblinded assessments and warrants further investigation. Nevertheless, we recommend clear, upfront expectation management to align patient perceptions with objective predicted outcomes.

Potential sources of bias were identified and considered, including selection bias, observer bias, and missing data. To minimize selection bias, all patients who received at least one treatment were included in the analysis, allowing assessment of immediate treatment‐related effects.

As this was a retrospective study, clinical data was not always complete for some patients. This limitation was considered in statistical analysis. Specifically, while a total of *n* = 93 patients were included in the study population, pre‐ and posttreatment photographic documentation suitable for efficacy evaluation was available for *n* = 27 patients (29%). All included patients were nevertheless assessed for safety outcomes.

Observer bias was mitigated through the study methodology, as evaluators assessing treatment efficacy based on photographic documentation were blinded to treatment details.

### 4.1 Limitations

Several limitations should be considered when interpreting these results. The retrospective nature of the study may introduce selection bias within the patient group. Differences in correlations between outcome measures could be due to varying expectations between patients and physicians, biases from assessments that were not blinded, or limitations due to small sample sizes for different evaluation methods. The availability of outcome measures varied widely among patients, potentially affecting the results and introducing reporting bias. The VISIA analysis was particularly constrained by a very small sample size, making interpretations of this measure potentially underpowered to detect significant associations; results from this patient cohort are interpreted as trends. Furthermore, the follow‐up was quite short due to the nature of the study. Neocollagenesis requires about 2–4 weeks to be recognizable in tightened skin and less wrinkles and usually it takes about 4–6 months to become fully apparent [27]. However, due to the short follow‐up time in this study, it can be speculated that the clinical outcome might improve with longer observation. An enhancement of clinical results can be achieved by repeating treatments as demonstrated in this study. Hence, it is in general recommended to repeat the treatment about 4 times with a short treatment pause of about 1–2 weeks. Future prospective, randomized, and controlled studies, with standardized assessment protocols, systematic follow‐up procedures, and larger sample sizes are needed to substantiate these results.

## 5 Conclusions

This study demonstrates that treatment with the unipolar RF device applicator improved skin laxity and reduced the severity of facial wrinkles across multiple validated assessment measures. To maximize patient satisfaction, clinicians should establish realistic expectations early in the consultation process, aligning them with objective and predictable clinical outcomes.

## Author Contributions

Krenar Dobroshi: data curation, writing – original draft preparation, clinical data selection. Krenar Dobroshi, Iva Stoilova: clinical data selection, manuscript review. Bekim Ismaili: data curation, manuscript review. Marija Glavash Dodov: data curation, manuscript review. Renata Slaveska Raichki: data curation, manuscript review.

## Funding

The authors have nothing to report.

## Ethics Statement

Reviewed and approved by local ethical committee Alma European Campuc College “Rezonanca” (No AD‐5025/24).

## Consent

The authors have nothing to report.

## Conflicts of Interest

The authors declare no conflicts of interest.

## Data Availability

Research data are not shared.
